# The Relationship of Fatty Food Consumption, History of Disease, and Physical Activity With the Event of Adolescent Obesity in Indonesia

**DOI:** 10.1155/jnme/2693160

**Published:** 2026-07-28

**Authors:** Julhan Irfandi, Marlina Josefina Malisngorar, Daniela L. Adeline Boeky, Sarci Magdalena Toy, Ali Muthahhari Rahim, Elmerilia Tandilangi, Farahdila Mirshanti, Ummi Kalsum, Adelina Fitri, Dewi Syafitriani, Nirmin F. Juber, Bhisma Murti

**Affiliations:** ^1^ Master Program of Public Health, Faculty of Medicine, Universitas Sebelas Maret, Surakarta, Indonesia, uns.ac.id; ^2^ Doctoral Program of Public Health, Faculty of Medicine, Universitas Sebelas Maret, Surakarta, Indonesia, uns.ac.id; ^3^ Doctoral Program of Public Health, Faculty of Public Health, Universitas Diponegoro, Semarang, Indonesia, undip.ac.id; ^4^ Bachelor of Public Health, Faculty of Medicine and Health Science, Universitas Jambi, Jambi, Indonesia; ^5^ Department of Health Promotion and Education, National Taiwan Normal University, Taipei, Taiwan, ntnu.edu.tw; ^6^ Doctoral Program of Public Health, Postgraduate School, Universitas Strada Indonesia, Kediri, Indonesia

**Keywords:** adolescents, fatty foods, infectious diseases, obesity, physical activity

## Abstract

**Background:**

This study examines the prevalence and associated factors of adolescent obesity using data from the 2018 Basic Health Survey, which includes 75.800 Indonesian adolescents aged 13–18 years.

**Methods:**

This study used cross‐sectional data from the 2018 Basic Health Survey, which involved 75.800 adolescents aged 13–18 years. Bivariate logistic regression was performed to estimate prevalence odds ratios (ORs) and 95% confidence intervals for factors associated with adolescent obesity. Multiple logistic regression was used to analyze the relationships between obesity, consumption of fatty foods, disease history, physical activity, age, gender, place of residence, parental history of overweight, and maternal occupation.

**Results:**

It reports 6.2% prevalence of obesity among adolescents and identifies key influencing factors, such as frequent consumption of fatty foods, history of disease, moderate physical activity levels, age, gender, place of residence, and parental history of overweight status. The strongest predictor of adolescent obesity is identified as a parental history of overweight (aOR = 4.45).

**Conclusions:**

This study concluded that reducing high‐calorie food consumption and promoting increased physical activity are crucial, particularly for adolescents with a familial history of being overweight. Overall, this research makes a significant contribution to understanding the prevalence and determinants of adolescent obesity in Indonesia. Its large dataset and comprehensive analysis are notable strengths, providing valuable insights for public health interventions.

## 1. Introduction

Nutrition plays a major role in shaping the productivity and quality of human resources; therefore, adequate nutritional intake is essential to meet physiological needs. However, inappropriate nutritional intake may lead to nutritional problems. To date, undernutrition remains unresolved, while overnutrition, characterized by overweight and obesity, continues to increase globally and nationally [1]. This condition reflects the double burden of malnutrition, which affects various age groups, including adolescents, particularly in low‐ and middle‐income countries undergoing rapid nutritional transition [1, 2]. Despite this broad understanding, the current discourse often remains descriptive and fails to clearly identify which dimension of the nutritional transition constitutes the most urgent public health problem, particularly in rapidly developing countries such as Indonesia.

Adolescence is a critical period of growth and development that significantly influences health outcomes in later life. It has been reported that approximately 80% of obese adolescents are likely to remain obese in adulthood [2], highlighting the long‐term implications of early‐life obesity. Previous studies have shown that obesity is more prevalent in mid‐to‐late adolescence (15–17 years) [3], and age has been identified as a significant factor associated with obesity risk [4]. Overweight during adolescence is therefore a crucial determinant of adult nutritional status and chronic disease risk [5]. Recent global evidence further confirms that adolescent obesity is strongly associated with long‐term cardiometabolic disorders, including type 2 diabetes and cardiovascular disease [6].

However, while these findings establish the importance of adolescence as a critical window, they do not sufficiently explain how these risks manifest differently across population contexts, particularly in countries experiencing rapid socioeconomic and lifestyle transitions. Obesity among adolescents has become a major public health concern worldwide, including in Indonesia. Data from the Global Nutrition Report indicate a substantial and increasing burden of adolescent obesity globally.

The impact of obesity extends beyond individual health outcomes and increases in the prevalence of overweight and obesity among children and adolescents aged 5–19 years [7]. In Southeast Asia, UNICEF reported that Indonesia ranks among countries with relatively high adolescent obesity prevalence [8]. National data also show an increase in obesity among adolescents aged 13–18 years, rising from 4.1% in 2013 to 8.8% in 2018 [9]. This near doubling within 5 years reflects a rapid epidemiological transition and signals a shift in Indonesia’s nutritional challenges toward overnutrition. More importantly, this trend indicates that existing nutrition policies historically focused on undernutrition may not be adequately equipped to address the rising burden of adolescent obesity [8]. The absence of comprehensive national‐level evidence on key determinants further constrains the effectiveness of current prevention strategies. Given Indonesia’s large adolescent population, this issue poses a serious challenge to achieving the demographic dividend and strengthening future human capital [10].

The findings highlight the broader impact of TB on the overall health system burden. Obesity is associated with an increased risk of chronic diseases such as hypertension, cardiovascular disease, stroke, cancer, and type 2 diabetes, as well as musculoskeletal disorders [1, 2]. It also contributes to increased healthcare costs and reduced productivity. Furthermore, adolescents with obesity often experience multiple comorbidities that are underdiagnosed and inadequately managed, including psychosocial and mental health consequences. Contemporary evidence highlights that obesity in adolescence is a complex condition involving biological, behavioral, and environmental pathways [11]. Previous studies have identified various determinants of adolescent obesity. Direct factors include dietary intake and medical history, while indirect factors include lifestyle behaviors, physical activity, environmental conditions, and psychosocial influences [7]. Several studies have shown that high consumption of fatty foods and fast food significantly increases the risk of obesity in adolescents [12–14], while sedentary behavior and low physical activity also contribute substantially [15]. However, recent high‐impact research suggests that obesity cannot be explained solely by lifestyle factors, but rather results from complex interactions between behavioral, environmental, and familial determinants [4, 11]. In particular, increasing attention has been given to the role of parental obesity as a key determinant of adolescent obesity. Evidence indicates that adolescents with obese parents have approximately 2‐3 times higher risk of obesity, reflecting the combined influence of genetic inheritance, epigenetic mechanisms, and shared household environments, including dietary patterns and physical activity behaviors [14, 16]. This indicates that obesity risk is embedded within family systems, yet this dimension remains insufficiently integrated into many epidemiological analyses [17].

Despite the growing body of research, several critical gaps remain. First, many studies in Indonesia are limited to local or school‐based populations, reducing generalizability to the national level [18]. Second, previous research often examines behavioral, environmental, and familial factors separately, without integrating these determinants into a comprehensive analytical framework. Third, findings regarding dominant risk factors remain inconsistent, particularly concerning the relative contribution of lifestyle versus familial influences [15]. These limitations hinder the development of evidence‐based and targeted public health policies for adolescent obesity prevention in Indonesia [16–18].

Therefore, this study aims to analyze the prevalence and determinants of adolescent obesity in Indonesia using nationally representative data. Unlike previous studies, this research applies an integrated analytical approach that simultaneously examines behavioral, demographic, and familial variables, with a particular focus on parental obesity. By identifying the most influential determinants at the population level, this study provides new, policy‐relevant evidence to support more effective and targeted interventions for adolescent obesity prevention and control in Indonesia.

## 2. Materials and Methods

### 2.1. Study Design and Data Source

This study employed an analytic cross‐sectional design using secondary data from the 2018 Indonesian Basic Health Survey (Riskesdas), a nationally representative survey conducted by the Ministry of Health of Indonesia. The cross‐sectional design was selected to assess the association between multiple risk factors and adolescent obesity at the population level within a specific time period. Riskesdas 2018 utilized a two‐stage stratified cluster sampling design based on the national population census framework, ensuring representativeness at both national and regional levels. Despite its strengths, this design has limitations, including the inability to establish causal relationships and the possibility of temporal ambiguity between exposure and outcome variables.

### 2.2. Study Population and Sample

The study population consisted of adolescents aged 13–18 years who participated in Riskesdas 2018. The analytical sample was obtained through a structured selection process based on predefined inclusion and exclusion criteria. Adolescents with complete data for all study variables were included. Participants were excluded if they were married, had incomplete or invalid anthropometric data (body mass index [BMI]‐for‐age), or had missing or inconsistent information on key exposure variables. Additionally, individuals with implausible nutritional status classifications were excluded to reduce the risk of misclassification bias. After applying these criteria, a total of 54,910 adolescents were included in the final analysis. These selection procedures were implemented to *ensure data quality and enhance the internal validity of the study*.

### 2.3. Variable Measurement

The dependent variable in this study was nutritional status, specifically obesity, measured using BMI‐for‐age based on the WHO AnthroPlus standard. Anthropometric data, including weight and height, were collected by trained enumerators using standardized measurement procedures. Nutritional status was categorized according to WHO growth reference standards as normal (−2 SD to +1 SD) and obese (> +2 SD). The independent variables included fatty food consumption, history of disease, and physical activity. Fatty food consumption was assessed using a structured questionnaire from Riskesdas 2018, which measured the frequency of consuming high‐fat foods such as fried foods and fast food. Responses were categorized as frequent, occasional, and rarely or never. History of disease was obtained from self‐reported questionnaire data regarding the occurrence of infectious or other relevant diseases within the past 1‐2 years and categorized as “ever had a disease” or “never had a disease.” Physical activity was measured based on the intensity and duration of daily activities using standardized Riskesdas instruments and categorized into light, moderate, and vigorous levels. Covariates included age group (13–15 years and 16–18 years), sex (male and female), place of residence (urban and rural), parental history of overnutrition (yes and no), and maternal employment status (working and not working). All variables were derived from standardized Riskesdas questionnaires and categorized according to national guidelines and relevant literature.

### 2.4. Data Collection and Instrument Validity

All data were collected through standardized household and individual questionnaires as part of Riskesdas 2018 within a defined national data collection period. The survey employed trained enumerators and calibrated measurement tools to ensure consistency and accuracy. The instruments used in Riskesdas 2018 had undergone prior validity and reliability testing, as documented in official reports from the Ministry of Health. Standardized protocols for anthropometric measurements and questionnaire administration were applied to maintain data quality and comparability across study locations.

### 2.5. Statistical Analysis

Data analysis was conducted using a stepwise approach, including univariate, bivariate, and multivariable analyses. Univariate analysis was performed to describe the distribution of all study variables. Bivariate analysis was conducted using logistic regression to estimate the association between independent variables and obesity, expressed as prevalence odds ratios (PORs) with 95% confidence intervals (CIs). Multivariable logistic regression analysis was then performed to assess the independent associations between exposure variables and obesity, controlling for potential confounders. Logistic regression was chosen because the outcome variable was dichotomous, making it appropriate for estimating associations in cross‐sectional studies. Confounding was addressed by including theoretically relevant covariates in the final model based on epidemiological considerations and prior evidence. Model fit was evaluated using the omnibus test to assess overall model significance, and pseudo *R* ^2^ statistics (Cox and Snell and Nagelkerke) were used as indicators of model fit rather than direct measures of explained variance. A *p* value of less than 0.05 was considered statistically significant.

### 2.6. Ethical Considerations

This study utilized secondary data from the 2018 Indonesian Basic Health Survey (Riskesdas 2018). Ethics and permissions for conducting this study followed the ethical approval for Riskesdas 2018 from the Health Research Ethics Committee, National Institute of Health Research and Development (NIHRD), Ministry of Health, Republic of Indonesia (Approval Number: LB.02.01/2/KE.267/2017). Permission to access and use the dataset for research purposes was officially granted by the Ministry of Health, Republic of Indonesia. All data analyzed in this study were fully anonymized, and no personally identifiable information was accessible to the researchers. Therefore, this study ensured confidentiality and complied with ethical standards for research involving human subjects.

## 3. Results

### 3.1. Univariate Analysis

Description of the participants: A total of 54,910 Indonesian adolescents aged 13–18 years participated in this study. The prevalence of obesity among the participants was 6.2%, while the majority (93.8%) were classified as nonobese. More than half of the adolescents (52.7%) were in the 13–15‐year age group, and 47.8% were aged 16–18 years. In terms of gender, the sample was almost evenly distributed, with males accounting for 51.5% and females for 48.5%. Most participants resided in urban areas (53.9%), while 46.1% lived in rural settings. Regarding parental characteristics, 69.9% of the adolescents had parents with a history of overweight, and 51.4% reported that their mothers were employed. In terms of dietary habits, 44.9% of the adolescents reported often consuming fatty foods, 45.1% consumed them sometimes, and 10.0% rarely or never consumed them.

### 3.2. Bivariate Analysis Association Between Consumption of Fatty Foods, History of Disease, Physical Activity, and Characteristics With the Incidence of Adolescent Obesity

Table 1 (bivariate analysis) shows several factors significantly associated with adolescent obesity. Significant factors include food consumption, history of disease, age, sex, place of residence, parental history of overweight status, and physical activity. Adolescents who *often* consumed fatty foods were at higher risk than those who *rarely or never* consumed them (OR: 1.29; 95% CI: 1.07 to 1.55; *p* = 0.006). *Sometimes* consumption also increased the risk of obesity, although less than *often* (OR: 1.23; 95% CI: 1.02 to 1.48; *p* = 0.024). Adolescents with *any* history of disease had a higher risk than those with *no history* (OR: 1.31; 95% CI: 1.12–1.52; *p* < 0.001). Adolescents aged 13–15 years were at higher risk than those aged 16–18 years (OR: 1.43; 95% CI: 1.29 to 1.59; *p* < 0.001). Male adolescents had a higher risk than females (OR: 1.14; 95% CI: 1.03 to 1.27; *p* = 0.015). Those living in urban areas had a higher risk than those in rural areas (OR: 1.67; 95% CI: 1.50 to 1.86; *p* < 0.001). A parental history of overweight was the most influential factor, with a 4.45‐fold higher risk among those with such a history compared to those without (OR: 4.45; 95% CI: 3.76–5.27; *p* < 0.001). Adolescents with *average* physical activity had a higher risk than those with *heavy* activity (OR: 1.20; 95% CI: 1.04 to 1.37; *p* = 0.012), while *lightweight* physical activity was not significant (OR: 1.12; 95% CI: 0.98 to 1.28; *p* = 0.108). Maternal employment status showed no significant association, with an OR of 0.99 (95% CI: 0.89 to 1.11; *p* = 0.930). Overall, adolescent obesity was significantly associated with food consumption, history of disease, age, sex, place of residence, parental overweight history, and average physical activity, while other factors were not significant.

**TABLE 1:** Bivariate analysis of fatty food consumption, history of disease, physical activity, and characteristics with the incidence of adolescent obesity.

Variable	Obesity						Odds ratios (95% CI)	*p* value
	Yes		No					
	*n*	%	*n*	%	*n*	%		
*Fatty food consumption*								
Often	1601	6.5	23,049	93.5	24,650	100	1.29 (1.07–1.55)	0.006
Sometime	1538	5.2	23,222	93.8	24,760	100	1.23 (1.02–1.48)	0.024
Rarely or never	281	5.1	5219	94.9	5500	100	Ref	

*History of disease*								
Any	475	7.7	5669	92.3	6145	100	1.31 (1.12–1.52)	< 0.001
Nothing	2945	6.0	45,821	94.0	48,765	100	Ref	

*Age*								
13–15 years	2065	7.2	26,572	92.8	28,637	100	1.43 (1.29–1.59)	< 0.001
16–18 years	1354	5.2	24,918	94.8	26,273	100	Ref	

*Sex*								
Male	1866	6.6	26,397	93.4	28,262	100	1.14 (1.03–1.27)	0.015
Female	1554	5.8	25,094	94.2	26,648	100	Ref	

*Place of residence*								
Urban	2238	7.6	27,353	92.4	29,591	100	1.67 (1.50–1.86)	< 0.001
Rural	1182	4.7	24,137	95.3	25,319	100	Ref	

*Parent’s history of overweight status*								
Yes	3099	8.1	35,268	92.6	38,367	100	4.45 (3.76–5.27)	< 0.001
No	321	1.9	16,222	98.1	16,543	100	Ref	

*Physical activity*								
Lightweight	1441	5.3	21,549	93.7	22,990	100	1.12 (0.98–1.28)	0.108
Average	1130	6.7	15,780	93.3	16,910	100	1.20 (1.04–1.37)	0.012
Heavy	849	5.7	14,611	94.3	15,010	100	Ref	

*Employment of mother*								
Work	1754	6.2	26,473	93.8	8227	100	0.99 (0.89–1.11)	0.930
No work	1666	6.2	25,017	93.8	26,682	100	Ref	

*Note:* Source: Secondary data processed, 2018.

### 3.3. Multivariate Analysis: Dominant Factors in the Incidence of Adolescent Obesity in Indonesia

Table 2 (multivariate logistic regression) identifies key factors associated with adolescent obesity in Indonesia. Among all variables, parental history of overweight was the most dominant factor, increasing the risk of obesity by 4.2 times compared to adolescents whose parents were not overweight (aOR = 4.20; 95% CI: 3.54 to 4.97; *p* < 0.001), after adjusting for age, sex, place of residence, fatty food consumption, history of disease, and physical activity. Other variables significantly associated with obesity were history of disease (aOR = 1.28; 95% CI: 1.09 to 1.50; *p* = 0.002), age 13–15 years (aOR = 1.44; 95% CI: 1.30 to 1.61; *p* < 0.001), male sex (aOR = 1.16; 95% CI: 1.04 to 1.29; *p* = 0.009), and urban residence (aOR = 1.46; 95% CI: 1.32 to 1.63; *p* < 0.001). In contrast, fatty food consumption (aOR = 1.19; 95% CI: 0.99 to 1.44; *p* = 0.175) and physical activity (aOR = 1.01; 95% CI: 0.88 to 1.16; *p* = 0.205) were not significantly associated with obesity. The model was statistically valid with an omnibus *p* value < 0.001 and an overall classification accuracy of 93.8%, while the Nagelkerke *R* ^2^ was 0.057, indicating that the independent variables explained 5.7% of the variability in adolescent obesity, and the remaining 94.3% was attributed to other unmeasured factors.

**TABLE 2:** Multivariate analysis of factors associated with adolescent obesity in Indonesia.

Variable	B	aOR (95% CI)	*p* value
Fatty food consumption	0.176	1.19 (0.99–1.44)	0.175
	0.150	1.16 (0.97–1.40)	

History of disease	0.247	1.28 (1.09–1.50)	0.002

Physical activity	0.009	1.01 (0.88–1.16)	0.205
	0.108	1.11 (0.97–1.28)	

Age	0.369	1.44 (1.30–1.61)	< 0.001

Sex	0.144	1.16 (1.04–1.29)	0.009

Place of residence	0.381	1.46 (1.32–1.63)	< 0.001

Parents’ history of overweight status	1.434	4.20 (3.54–4.97)	< 0.001

Omnibus *p* value			< 0.001

Overall percentage			93.8

*Note:* Source: Secondary data processed, 2018.

Abbreviations: aOR = adjusted odds ratio; CI = confidence interval.

## 4. Discussion

Based on the results of this study, the prevalence of adolescent obesity in Indonesia was 6.2%, with higher proportions among adolescents exhibiting unhealthy dietary patterns, disease history, and parental overweight. In the multivariable model, parental history of overweight emerged as the strongest determinant (aOR = 4.20), followed by urban residence, age, sex, and disease history. These findings highlight that adolescent obesity is not solely a behavioral issue but a multifactorial condition shaped by biological, environmental, and social interactions [10, 12, 19].

### 4.1. Association Between Consumption of Fatty Foods and Adolescent Obesity

Fatty food consumption was associated with obesity in bivariate analysis (OR = 1.29) but lost significance in multivariate analysis (aOR = 1.19; 95% CI: 0.99 to 0.44; *p* = 0.175). This suggests that dietary intake alone does not independently explain obesity risk. Recent studies highlight that high‐fat diets are embedded in broader clusters of obesogenic behaviors, including low physical activity and high sedentary time, which collectively increase obesity risk [18, 20]. Biologically, excessive fat intake promotes energy storage, insulin resistance, and adiposity, yet these effects are strongly influenced by environmental food availability, cultural eating patterns, and social norms. Therefore, dietary behavior should be interpreted as part of a broader obesogenic system rather than an independent risk factor [21, 22].

### 4.2. Association Between Disease History and Adolescent Obesity

Adolescents with a history of disease had a higher obesity risk (aOR = 1.28; 95% CI: 1.09 to 1.50; *p* = 0.002). Illness can reduce physical activity, increase sedentary behavior, and elevate caloric intake during recovery, contributing to weight rebound, especially after infectious or chronic diseases. Conversely, obesity predisposes adolescents to metabolic and inflammatory disorders, creating a bidirectional relationship [20]. These findings underscore the need for public health strategies integrating disease management, post‐recovery nutritional guidance, and behavioral support to mitigate obesity risk, highlighting the complex interplay between health status and weight outcomes [11, 22, 23].

### 4.3. Association Between Physical Activity and Adolescent Obesity

Physical activity was significant in bivariate analysis but not in multivariate analysis (aOR = 1.01; 95% CI: 0.88 to 1.16; *p* = 0.205), suggesting that activity alone is insufficient to explain obesity risk without considering diet, sedentary behavior, and environmental factors [24]. Global evidence indicates insufficient physical activity remains a major contributor to adolescent obesity, but its effect is highly context‐dependent. Sedentary behavior, including prolonged screen time, independently increases obesity risk even among active adolescents, emphasizing the need for integrated interventions targeting multiple lifestyle domains [25, 26].

### 4.4. Dominant Factors Associated With Adolescent Obesity

Parental history of overweight was the strongest predictor of adolescent obesity (aOR = 4.20; 95% CI: 3.54 to 4.97; *p* < 0.001), reflecting genetic susceptibility and shared family environment, including dietary habits and physical activity patterns [27–29]. Epigenetic evidence suggests parental metabolic status influences offspring adiposity through genetic and environmental pathways [30, 31]. Urban residence was also significantly associated (aOR = 1.46; 95% CI: 1.32 to 1.63; *p* < 0.001), consistent with environments offering high‐calorie foods, reduced green spaces, and sedentary lifestyles [31, 32]. These findings support a systems‐level understanding, where familial predisposition interacts with urban obesogenic environments to shape adolescent obesity [33, 34].

These findings underscore that adolescent obesity should be addressed through comprehensive, multilevel strategies. Given that parental overweight emerged as the most dominant factor, public health interventions should prioritize family‐centered approaches, including parental nutrition education, promotion of healthy household behaviors, and routine monitoring of child growth and parental risk factors [35–37]. Integrating parental screening into school‐based health programs and strengthening community‐based interventions may enhance early identification and prevention efforts. In addition, promoting active transportation can improve health outcomes and reduce the overall burden on the health system. [38–40].

However, several limitations should be considered when interpreting these findings. The cross‐sectional design of this study limits the ability to establish causal relationships between identified risk factors and adolescent obesity. Additionally, the use of self‐reported data on dietary intake and physical activity may introduce recall and social desirability bias. Potential confounding variables, such as sleep patterns, psychological stress, and exposure to food marketing, were not included in the analysis and may influence the observed associations. Despite these limitations, the study provides important insights into the multifactorial nature of adolescent obesity.

## 5. Conclusion

This study demonstrates that adolescent obesity in Indonesia is a multifactorial condition shaped by the interaction of biological, environmental, and behavioral factors. Parental history of overweight emerged as the most dominant determinant, highlighting the critical role of genetic susceptibility and shared family environments. The findings further indicate that lifestyle factors such as diet and physical activity do not operate independently but are embedded within broader familial and environmental contexts, particularly in urban settings that promote obesogenic behaviors.

These results underscore the need to shift from individual‐based approaches toward integrated, multilevel strategies that prioritize high‐risk groups, especially adolescents with parental overweight in urban areas. Future research should focus on longitudinal designs and the exploration of gene–environment interactions to better understand causal mechanisms and improve intervention targeting. From a policy perspective, existing national programs such as the Gerakan Nusantara Tekan Angka Obesitas (GENTAS) should be strengthened by incorporating targeted, family‐based strategies, including parental risk screening, early identification through school‐based programs, and tailored behavioral interventions for high‐risk households. Integrating these approaches with urban environmental policies, such as improving access to recreational spaces and regulating unhealthy food environments, may enhance the effectiveness of obesity prevention efforts.

## Author Contributions

Julhan Irfandi was responsible for conceptualization, methodology, formal analysis, data curation, data processing and analysis, writing the original draft, and project administration. Daniela L. Adeline Boeky and Marlina Josefina Malisngorar contributed to methodology, validation, writing–review and editing, and visualization. Sarci Magdalena Toy, Dewi Syafitriani, and Elmerilia Tandilangi contributed to data curation, investigation, and writing–review and editing. Farahdila Mirshanti contributed to the investigation, visualization, and writing–review and editing. Bhisma Murti, Nirmin F. Juber, Ummi Kalsum, and Adelina Fitri provided supervision and contributed to the critical review and editing of the manuscript. Ali Muthahhari Rahim contributed to the revision process, particularly in strengthening and refining the physical activity variable in accordance with his subject matter expertise.

## Funding

This research was funded by the Indonesia Endowment Funds for Education (LPDP), Lembaga Pengelola Dana Pendidikan, Ministry of Finance of the Republic of Indonesia, through scholarship and research funding programs.

## Disclosure

All authors have read and approved the final version of the manuscript.

## Ethics Statement

This study was conducted using anonymized secondary data from the Basic Health Survey 2018 with approval from the relevant ethics committee of the Indonesian government. All participants consented to participate in this study.

## Consent

Written informed consent was obtained from all participants before the survey.

All authors consent to publication.

## Conflicts of Interest

The authors declare no conflicts of interest.

## Data Availability

The data used in this study are secondary data obtained from the 2018 Basic Health Survey conducted by the National Institute of Health Research and Development, Ministry of Health of the Republic of Indonesia. Access to these data is restricted and can be obtained through an official request to the Basic Health Survey data management team, subject to their terms and conditions. The authors do not have the authority to share the data directly. Requests for access to the data should be directed to the official website of the National Institute of Health Research and Development, Ministry of Health, Republic of Indonesia.
